# Mutational landscape of classical myeloproliferative neoplasms in the Western Cape Province, South Africa

**DOI:** 10.4102/ajlm.v15i1.2965

**Published:** 2026-08-20

**Authors:** Marthinus J. Dicks, Erica-Mari Nell, Carmen Swanepoel, Ibtisam Abdullah, Zivanai C. Chapanduka

**Affiliations:** 1 Department of Pathology, Faculty of Medicine and Health Sciences, Stellenbosch University, Cape Town, South Africa; 2 Division of Haematology, Department of Pathology, Health New Zealand, Northern Region, Whangarei, New Zealand

**Keywords:** polycythaemia vera, essential thrombocythaemia, primary myelofibrosis, myeloproliferative neoplasms, mutational landscape

## Abstract

**Background:**

The classical myeloproliferative neoplasms (MPNs) are driven by somatic variants in *Janus kinase 2* (*JAK2*), *calreticulin* (*CALR*) and *thrombopoietin receptor* (*MPL*) genes. The heterogeneity in mutational frequency of *JAK2, CALR, MPL* and triple-negative MPNs between regions indicates that epidemiological studies in sub-Saharan Africa are required.

**Objective:**

The aim of this study was to describe the genetic variants seen in MPNs at a South African tertiary hospital.

**Methods:**

A retrospective study was conducted at Tygerberg Hospital, Western Cape Province, South Africa. Patients investigated for a classical MPN driver variant (polycythaemia vera [PV], primary myelofibrosis [PMF] and essential thrombocythaemia [ET]) from 2009 to 2020 were included in the study. The associated MPN diagnosis was sought from full blood count and bone marrow reports. *Janus kinase 2*-positive and -negative groups were compared for each MPN using a two-tailed independent samples *t*-test.

**Results:**

There were 128 patients diagnosed with an MPN over the 12-year study period. Polycythaemia vera was the most prevalent (38%), followed by PMF (33%), essential thrombocythaemia (20%), and MPN unclassifiable (9%). The most frequent variant was *JAK2* p.V617F (78%) followed by *CALR* variants (8%). No *MPL* variants were detected among the patients tested. In PMF, patients with *JAK2* p.V617F variants were older (mean age 65 years vs 58 years, *p* = 0.048) and had a higher haemoglobin (10.6 g/dL vs 8.1 g/dL, *p* = 0.013) at diagnosis when compared to patients without *JAK2* p.V617F.

**Conclusion:**

These data suggest that there are differences with regard to MPN epidemiology and variant frequency in South Africa, and further clinical studies are required to fully characterise MPNs in the South African context.

**What this study adds:**

This retrospective study expanded the profiling of MPNs and driver variants of MPNs in a South African population. Primary myelofibrosis made up a higher percentage of MPN cases than reported in international studies. In addition, in PMF and essential thrombocythaemia, *JAK2* p.V617F was more common and *CALR* variants were less common than reported internationally, while *MPL* variants were not detected.

## Introduction

The classical myeloproliferative neoplasms (MPNs), namely polycythaemia vera (PV), essential thrombocythaemia (ET) and primary myelofibrosis (PMF), have overlapping clinical, morphological and molecular features. Primary myelofibrosis can be classified further into a pre-fibrotic and overt fibrotic stage. Cases with features of a MPN, which do not meet all criteria for a specific MPN entity, are classified as MPN unclassifiable.^1^

The canonical MPN driver gene variants include specific variants in *Janus kinase 2* (*JAK2*), *calreticulin* (*CALR*) and the *thrombopoietin receptor* gene (*MPL*).^2^ *Janus kinase 2* p.V617F accounts for 97% of PV and 55% of ET and PMF cases.^3^ Furthermore, *JAK2* exon 12 variants are reported in the majority of PV patients who are negative for the *JAK2* p.V617F variant.^4^ *Calreticulin* variants account for 25% of ET and 30% of PMF cases.^3^ *Calreticulin* variants can be subtyped as type 1 (52 bp deletion; c.1092_1143del52;p.Leu367Thrfs*46) and type 2 (5 bp insertion; c.1154_1155insTTGTC) *CALR* variants.^5^ In ET, type 1 and 2 *CALR* variants are more evenly distributed (51% for type 1 vs 39% for type 2) than in PMF (70% for type 1 vs 13% for type 2).^6^ *Thrombopoietin receptor gene* variants account for 5% of ET and 8% of PMF cases.^3^ The absence of *MPL* variants in some studies could indicate that *MPL* variants are not equally prevalent among all population groups.^7^

The absence of a classical driver gene variant in otherwise typical MPN suggests that there are alternative low-frequency non-canonical MPN variants which can result in a similar phenotype.^8,9^ Cases of triple-negative MPNs (negative for the canonical variants in *JAK2, CALR* and *MPL*) occur in ET and PMF in 10% to 15% of cases.^10^

Driver variants in *JAK2, CALR* and *MPL* affect the MPN phenotype and prognosis. Primary myelofibrosis patients with *JAK2* p.V617F are more likely to present with portal vein thrombosis, while those with *CALR* variants are younger, have a higher platelet count, and a lower leukocyte count when compared with other PMF patients.^11,12^ The risk of blast transformation in *CALR*-mutated PMF is lower than in *JAK2*-mutated or triple-negative PMF.^11,12^ There may be population-specific differences in the prognostic implications of a genetic driver. *Calreticulin*-mutated PMF was shown to have improved overall survival compared with *JAK2*-mutated PMF in the non-Asian population, while in the Asian group, *JAK2*-mutated PMF performed better.^13^ Such findings highlight the need for better description of the MPN mutational landscape in different populations.

The heterogeneity in variant frequency of *JAK2, CALR, MPL* and triple-negative MPNs between regions indicates that epidemiological studies in sub-Saharan Africa are required. The variant heterogeneity could be compounded in countries such as South Africa, where extensive genomic diversity is present.^14^ In addition, to our knowledge, there have been no published epidemiological studies assessing the frequency of *JAK2, CALR* or *MPL* variants in MPNs in South Africa. Understanding the genetic drivers has both diagnostic and therapeutic implications.

The aim of this study was, therefore, to describe the genetic variants found in MPNs in a South African population. The study objectives were to determine the frequency of PV, ET and PMF; to determine the frequency of *JAK2* p.V617F, *JAK2* exon 12, *CALR* and *MPL* variants in MPNs; and to compare full blood count parameters between variants within each MPN group.

## Methods

### Ethical considerations

This study was approved by the Human Research Ethics Committee of Stellenbosch University (Approval number: HREC1-2020-17308). A waiver of individual patient consent was granted because of the retrospective nature of the study and the fact that patients were de-identified. Collected data were entered on a REDCap® data entry sheet.^15^ Each data set was allocated a unique study number and de-identified for any subsequent analyses. The patient identifiers remained only on the REDCap® data entry sheet, which was protected with both a password and an encryption key. The anonymity of the patients was upheld throughout the study and no patient identifying information was made public during the data collection, data analysis, and final output of this study.

### Study setting

A retrospective cross-sectional descriptive study was conducted at Tygerberg Hospital, a tertiary academic hospital in South Africa. The hospital serves a catchment area of 3.4 million people. All sample testing was conducted at the National Health Laboratory Service.

### Data collection and interpretation

All patients aged 18 years or older who were investigated for the presence of a classical MPN variant (*JAK2* p.V617F, *JAK2* exon 12, *CALR* or *MPL* variants) at Tygerberg Hospital from January 2009 to December 2020 were included in the study. Patients who required a diagnostic bone marrow examination for MPN diagnosis, but did not have one, were excluded.

Patients were obtained from the database of patients tested for classical MPN variants at the Molecular Haematology Laboratory. The data extraction was performed between July and September 2021. Patient information was entered on a REDCap® data entry sheet.^15^ The National Health Laboratory Service Laboratory Information System was used to assess full blood count, erythropoietin, and bone marrow examination reports. Age at diagnosis and sex were also retrieved. Results from before August 2015 were obtained from the DisaLab Laboratory Information System (DisaLab, Laboratory System Technologies, Johannesburg, South Africa), while results from after August 2015 were obtained from the TrakCare Laboratory Information System (TrakCare, InterSystems Corporation, Cambridge, Michigan, United States).

*Janus kinase 2* variant testing for p.V617F and exon 12 variants commenced at Tygerberg Hospital in 2009, and *CALR* and *MPL* variant testing in 2015. The workflow for MPN testing at Tygerberg Hospital entailed first testing for *JAK2* p.V617F. If the result was negative, *JAK2* exon 12 variant testing was performed for patients with suspected PV, while for ET and PMF, *CALR* and *MPL* variant testing was performed. *Janus kinase 2* p.V617F testing was performed by allele-specific polymerase chain reaction. This method employs variant- and wild-type-specific forward primers with a common reverse primer. Genotype is determined by the presence or absence of the corresponding allele-specific amplicons. *Janus kinase 2* exon 12, *CALR* and *MPL* variant analysis was performed by bidirectional Sanger sequencing.

### Data analysis

Statistical analysis was performed using Microsoft Excel® (Microsoft, Redmond, Washington, United States). Results were summarised and analysed in association with the Biostatistics Unit, Division of Epidemiology and Biostatistics, at Stellenbosch University in South Africa. The Shapiro–Wilk test was used to determine whether the data deviated from a normal distribution. All variables (age at diagnosis, haemoglobin, white cell count, and platelet count) were parametric, and therefore mean and standard deviation were calculated. Variables were compared using the two-tailed independent samples *t*-test. The *JAK2* p.V617F-positive group was compared to the *JAK2* p.V617F-negative group for each classical MPN. For PV, the *JAK2* p.V617F-negative group consists of all patients negative for *JAK2* V617F. For ET and PMF, the *JAK2* V617F-negative group consists of *CALR* variant positive, *MPL* variant positive, and triple-negative patients. Pre-PMF and overt-PMF were analysed as one group (PMF). A *p*-value less than 0.05 was considered statistically significant.

## Results

**FIGURE 1:**
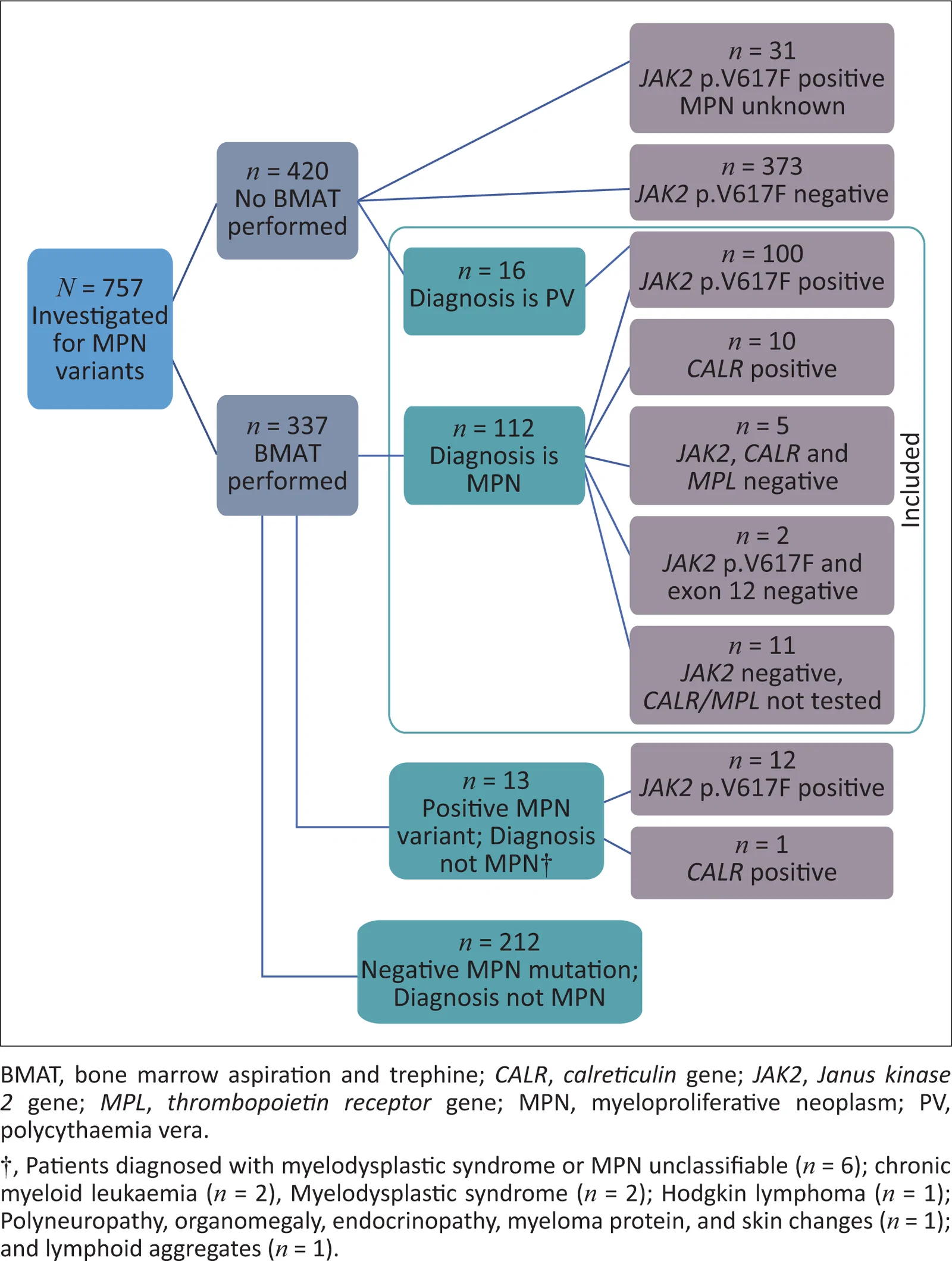
Summary of sample population by diagnosis and myeloproliferative neoplasm mutation status, Tygerberg Hospital, Cape Town, Western Cape, South Africa, January 2009 to December 2020.

A total of 757 patients were tested for variants in *JAK2* p.V617F, *JAK2* exon 12, *CALR* and/or *MPL* over the 12-year period (Figure 1). All patients were initially tested for *JAK2* p.V617F. Of these, 143 (19%) were positive for the variant and were not assessed for other variants based on the workflow of the testing laboratory.

Diagnostic bone marrow examination was not performed in 420 (55%) of the patients who had genetic analysis. Of these, 16 met the diagnostic criteria for PV without a bone marrow examination.^1^ Bone marrow examination was performed on 337 (45%) patients (Figure 1). In 63% of these (212/337), an MPN-associated variant was not detected and the final diagnosis on bone marrow examination was not in keeping with a classical MPN. In 4% (13/337), a variant for a classical MPN was detected; however, on bone marrow examination, the diagnostic criteria for a classical MPN were not met.

There were 128 patients diagnosed with an MPN and included for further analysis, with 16 of these meeting diagnostic criteria for PV without bone marrow aspiration and trephine (Figure 1). The average age of MPN diagnosis was 62 ± 14 years with a male to female ratio of 1:1 (64 male patients to 64 female patients). Of the 128 patients diagnosed with an MPN on bone marrow examination, 49 (39%) had PV, 26 (20%) had ET, 17 (13%) had pre-PMF, and 25 (19%) had overt-PMF. In addition, 11 patients (9%) were classified as MPN unclassifiable. The ages at presentation of PV, ET and PMF were similar (Table 1).

**TABLE 1:** Age and main haematological features of myeloproliferative neoplasm patients stratified by *Janus kinase 2* p.V617F status, Tygerberg Hospital, Cape Town, Western Cape, South Africa, January 2009 to December 2020.

MPN		*n*	Age (years)		White cell count (× 10^9^/L)		Haemoglobin (g/dL)		Platelet count (× 10^9^/L)	
			Mean ± s.d.	*p*	Mean ± s.d.	*p*	Mean ± s.d.	*p*	Mean ± s.d.	*p*
**PV**		49	62 ± 15	-	13.68 ± 7.77	-	16.6 ± 3.0	-	664 ± 411	-
**ET**		26	62 ± 16	0.145	12.5 ± 7.1	0.590	11.8 ± 2.7	0.135	1319 ± 793	0.437
	*JAK2* p.V617F positive	17	65 ± 14	-	12.9 ± 7.4	-	12.4 ± 2.8	-	1176 ± 468	-
	*JAK2* p.V617F negative	9	55 ± 17	-	11.8 ± 6.6	-	10.7 ± 2.5	-	1589 ± 1185	-
**PMF**		42	63 ± 12	0.048	22.8 ± 26.2	0.174	9.9 ± 3.1	0.013	620 ± 708	0.178
	*JAK2* p.V617F positive	30	65 ± 12	-	22.7 ± 21.8	-	10.6 ± 3.1	-	671 ± 754	-
	*JAK2* p.V617F negative	12	58 ± 10	-	22.9 ± 36.1	-	8.1 ± 2.1	-	494 ± 588	-

Note: PV *p*-values were not calculated because the *JAK2*-negative group had *n* = 2 as noted.

*CALR, calreticulin* gene; ET, essential thrombocythaemia; *JAK2, Janus kinase 2* gene; *MPL, thrombopoietin receptor* gene; MPN, myeloproliferative neoplasm; PMF, primary myelofibrosis; PV, polycythaemia vera.

**FIGURE 2:**
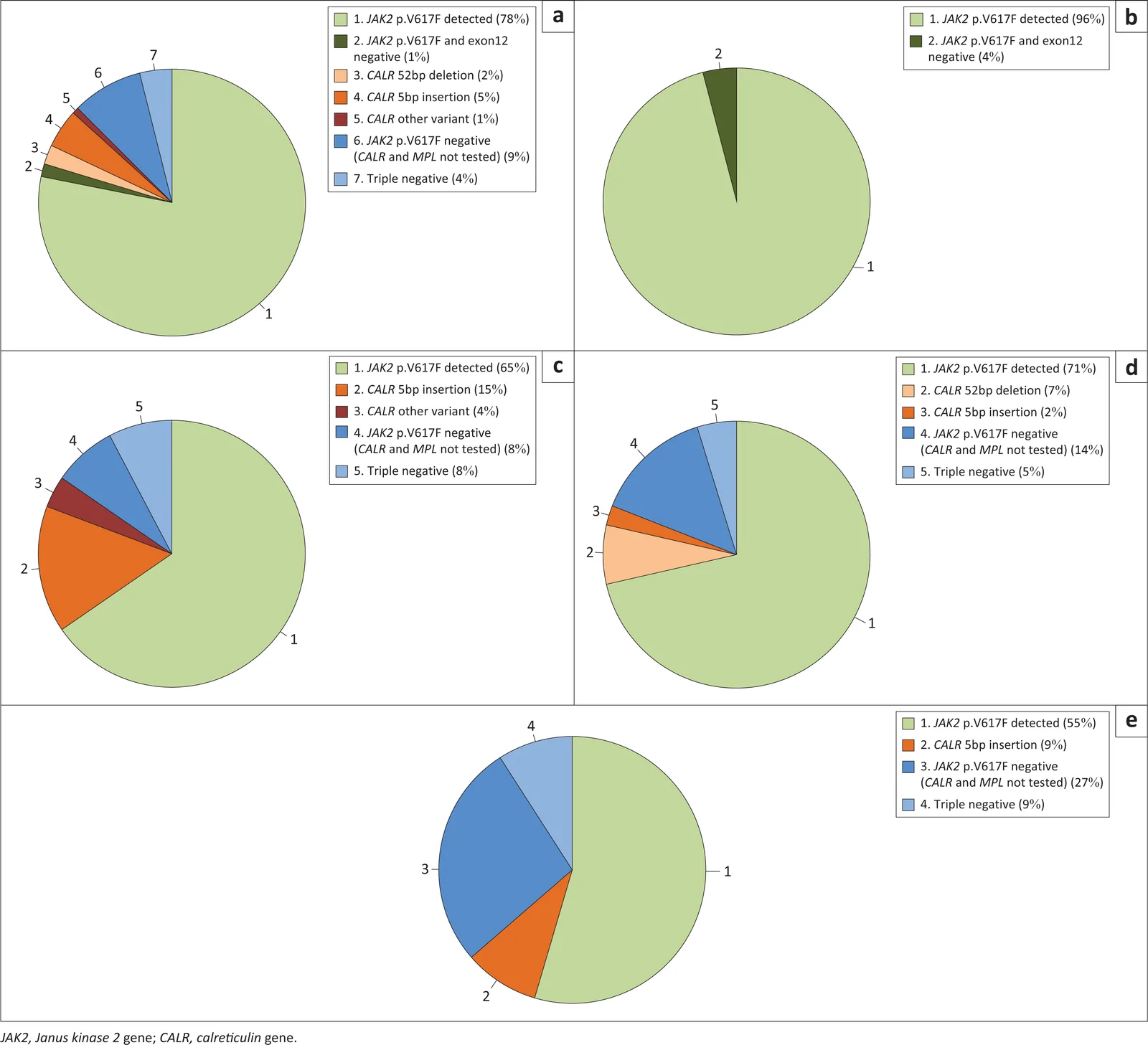
Genetic variant profile categorised by myeloproliferative neoplasm diagnoses for (a) all myeloproliferative neoplasms (*n* = 128); (b) polycythaemia vera (*n* = 49); (c) essential thrombocythaemia (*n* = 26); (d) primary myelofibrosis (*n* = 42); (e) myeloproliferative neoplasm unclassifiable (*n* = 11), Tygerberg Hospital, Cape Town, Western Cape, South Africa, January 2009 to December 2020.

In PV, *JAK2* p.V617F was seen in the majority of patients (96%). The remaining two (4%) *JAK2* p.V617F-negative patients with PV were also negative for *JAK2* exon 12 variants (Figure 2). The diagnosis of PV was based on the minor criterion of low erythropoietin level in these cases. In ET and PMF, the most common variant was *JAK2* p.V617F, seen in 65% (ET) and 71% (PMF) (Figure 2). Patients who were *JAK2* p.V617F negative were tested for *CALR* and *MPL* variants; that accounted for seven of the nine ET patients and six of the 12 PMF patients. The patients who were not tested for *CALR* or *MPL* variants were investigated for an MPN before testing for *CALR* and *MPL* variants became available at our centre. Three patients (2%) had a *CALR* 52 bp deletion (Type 1 mutation) – all of these were PMF cases. Five patients (5%) had a *CALR* 5 bp insertion (Type 2 mutation) – four were in patients with ET and the other one in a patient with PMF. A novel *CALR* frameshift variant was detected in one patient with ET [NM_004343.3(CALR):c.1104_1143del40 (p.E369Qfs)]. All tested patients were negative for an *MPL* variant. There were five triple-negative cases, two of which were ET, two PMF, and one case was MPN unclassifiable (Figure 2).

In PMF, haemoglobin was significantly higher in those patients positive for *JAK2* p.V617F compared to those negative for *JAK2* p.V617F (*p* = 0.013). In addition, patients diagnosed with PMF who lack the *JAK2* p.V617F variant were significantly younger than the *JAK2* p.V617F-positive group (*p* = 0.048). There were no other significant differences in ET or PMF between the *JAK2* p.V617F-positive and -negative groups (Table 1). There were too few PV patients who were negative for both *JAK2* p.V617F and exon 12 (*n* = 2) for meaningful comparison between groups.

## Discussion

We found that PV was the most common MPN subtype, accounting for 38% of MPNs, followed by PMF in 33%. Essential thrombocythaemia was the least common MPN, at 20%. We found that *JAK2* p.V617F was the most common variant across all MPNs, seen in 96% of PV, 65% of ET and 71% of PMF. No *MPL* variants were detected among the patients tested. In PMF, *JAK2* p.V617F was associated with older age at diagnosis and higher haemoglobin.

The proportion of PV, PMF and ET cases found in our study compared well to a seven-year retrospective study which examined *JAK2* p.V617F variants in the Gauteng Province of South Africa.^16^ Primary myelofibrosis was diagnosed in 43.7% of the Gauteng patients, and ET in 11.5%.^16^ In contrast, PMF is typically the least frequent of the three classical MPNs outside of South Africa.^7^ In a large meta-analysis of 52 studies, which included 5300 MPN patients from the United States, China, Brazil and Europe, only 18% of MPN cases were diagnosed with PMF and 49% with ET.^7^ The reason for this difference in the frequency of the MPNs between South African and international studies is unclear. It may reflect a true population-specific difference in MPN frequencies; however, it may also be that patients with PMF are more likely than those with ET both to seek medical care and to be referred from primary care facilities for further investigation because of the more pronounced symptom severity seen in PMF.

In our 49 patients with PV, 96% had the *JAK2* p.V617F. The frequency of *JAK2* p.V617F variant ranges from 37.8% to 100% in PV across different studies (Table 2). The average frequency of *JAK2* p.V617F-negative PV has been reported as 5% in literature from Europe and the United States, although the number varies slightly between studies (Table 2).^17^ This may be related to sensitivity of testing modality and sample study size, rather than differing driver variants. Of note, many studies did not perform a test for *JAK2* exon 12 variants. The only African studies investigating the frequency of *JAK2* exon 12 variants in PV patients were a Sudanese study that reported a *JAK2* exon 12 frequency of 8.1% and an Egyptian study with a frequency of 0% (Table 2).^18,19^ The frequency of PV driver variants in sub-Saharan African countries has not yet been determined. We only had two PV patients who were negative for *JAK2* p.V617F and both patients were negative for *JAK2* exon 12 variants. Further large-scale South African studies are required to more accurately characterise the frequency of *JAK2* exon 12 mutations within the local population.

**TABLE 2:** Driver variant frequencies in polycythaemia vera in this study compared with African and worldwide studies.

Continent	Studies investigating polycythaemia vera	*n*	Country	*JAK2* p.V617F detected (%)	*JAK2* exon 12 variant detected (%)
Africa	This study	49	South Africa	96	0
	Soliman et al.^18^	92	Egypt	48.9	0
	Eldeweny et al.^20^	14	Egypt	64.3	Not tested
	Ebid et al.^21^	70	Egypt	80	Not tested
	Abkar et al.^22^	159	Sudan	81.7	Not tested
	Ibrahim et al.^19^	83	Sudan	91	8.1
	Sassi et al.^23^	286	Tunisia	37.8	Not tested
	Benguella-Benmansour et al.^24^	98	West Algeria	81.6	Not tested
Rest of the world	Ojeda et al.^25^	176	Argentina	94.9	0
	Lin et al.^26^	234	China	85.0	4.3
	Wu et al.^27^	80	China	81.5	12.5
	Zhang et al.^28^	89	China	82	3.4
	Sazawal et al.^29^	34	India	82	Not tested
	Zulkeflee et al.^30^	76	Malaysia	86.8	Not tested
	Azevedo et al.^31^	39	Portugal	87.2	Not tested
	Yeh et al.^32^	22	Taiwan	77	23
	Lieu et al.^33^	33	Taiwan	85	Not tested
	Lee et al.^10^	48	United Kingdom, Italy	100	Not tested
	Pardanani et al.^34^	220	United States	97.2	2.3

Note: Please see the full reference list of Dicks MJ, Nell E-M, Swanepoel C, Abdullah I, Chapanduka ZC. Mutational landscape of classical myeloproliferative neoplasms in the Western Cape Province, South Africa. Afr J Lab Med. 2026;15(1), a2965. https://doi.org/10.4102/ajlm.v15i1.2965.

*JAK2, Janus kinase 2* gene.

Our findings showed that there were no *MPL* variants detected in the *JAK2* p.V617F-negative ET and PMF patients. The only other African studies, which were performed in Egypt, also failed to detect *MPL* variants in ET and PMF patients.^18,20^ Of note, in our study, there were 11 patients who did not undergo *MPL* testing after testing negative for *JAK2* p.V617F. Their mutational status is therefore unknown. Our findings support revising our institutional standard operating procedure to perform *JAK2* p.V617F testing upfront, given its high prevalence, followed by next-generation sequencing when *JAK2* p.V617F is negative. This strategy will improve detection of non-canonical mutations and, importantly, enhance identification of low allele frequency *CALR* variants that are currently under-recognised in South Africa.

*Janus kinase 2* p.V617F or *CALR* variants were present in the majority of ET and PMF patients in our study, a finding frequently reported in the literature (Table 3 and Table 4). The frequency of *CALR* variants in our study was on the lower end of the frequency distribution range, with the majority of our patients having a *JAK2* p.V617F variant. In our study, the prevalence of *JAK2* p.V617F is higher in PMF patients (71%) than ET patients (65%) (Table 3 and Table 4). *Calreticulin* 5 bp insertion mutations (*n* = 6) were more common in our patients than *CALR* 52 bp deletion mutations (*n* = 3), which contrasts with Europe (32% vs. 56%).^6^ There were only 10 *CALR* variant-positive patients in our study and larger studies are required to confirm this finding.

**TABLE 3:** Driver variant frequencies in essential thrombocythaemia in this study compared with African and worldwide studies.

Continent	Studies investigating essential thrombocythaemia	*n*	Country	*JAK2* variant (%)	*CALR* variant (%)	*MPL* variant (%)
Africa	This study	26	South Africa	65	19.2	0
	Soliman et al.^18^	68	Egypt	44.1	19.1	0
	Eldeweny et al.^20^	26	Egypt	Not stated	Not tested	0
	Ebid et al.^21^	24	Egypt	25	Not tested	Not tested
	Abkar et al.^22^	55	Sudan	56.4	Not tested	Not tested
	Sassi et al.^23^	275	Tunisia	44	Not tested	Not tested
	Benguella-Benmansour et al.^24^	75	West Algeria	58.7	Not tested	Not tested
Rest of the world	Ojeda et al.^25^	214	Argentina	61.2	21.5	6
	Klampfl et al.^5^	311	Austria, Italy	59.1	25.1	3.5
	Lin et al.^26^	428	China	58.4	22.7	1.2
	Wu et al.^27^	80	China	56.3	25	5
	Zhang et al.^28^	142	China	36.6	Not tested	1
	Sazawal et al.^29^	10	India	70	Not tested	Not tested
	Zulkeflee et al.^30^	41	Malaysia	70.7	7.3	0
	Azevedo et al.^31^	80	Portugal	73.4	Not tested	Not tested
	Lieu et al.^33^	49	Taiwan	59	Not tested	0
	Lee, Godfrey & Nangalia^10^	62	United Kingdom, Italy	56	29	8

Note: Please see the full reference list of Dicks MJ, Nell E-M, Swanepoel C, Abdullah I, Chapanduka ZC. Mutational landscape of classical myeloproliferative neoplasms in the Western Cape Province, South Africa. Afr J Lab Med. 2026;15(1), a2965. https://doi.org/10.4102/ajlm.v15i1.2965.

*CALR, calreticulin* gene; *JAK2, Janus kinase 2* gene; *MPL*, thrombopoietin receptor gene.

**TABLE 4:** Driver variant frequencies in primary myelofibrosis in this study compared with African and worldwide studies.

Continent	Studies investigating primary myelofibrosis	*n*	Country	*JAK2* variant (%)	*CALR* variant (%)	*MPL* variant (%)
Africa	This study	42	South Africa	71	9	0
	Soliman et al.^18^	40	Egypt	32.5	17.5	0
	Eldeweny et al.^20^	20	Egypt	30	Not tested	0
	Ebid et al.^21^	16	Egypt	12.5	Not tested	Not tested
	Abkar et al.^22^	45	Sudan	51.1	Not tested	Not tested
	Sassi et al.^23^	89	Tunisia	29.2	Not tested	Not tested
	Benguella-Benmansour et al.^24^	13	West Algeria	46.1	Not tested	Not tested
Rest of the World	Ojeda et al.^25^	49	Argentina	62	18	2
	Klampfl et al.^5^	203	Austria, Italy	53.2	35.4	6.4
	Lin et al.^26^	187	China	65.8	17.6	2.7
	Wu et al.^27^	50	China	58.0	36	6
	Zhang et al.^28^	47	China	51.1	Not tested	4
	Sazawal et al.^29^	31	India	52	Not tested	Not tested
	Zulkeflee et al.^30^	42	Malaysia	52.3	14.3	0
	Azevedo et al.^31^	14	Portugal	50.0	Not tested	Not tested
	Lieu et al.^33^	6	Taiwan	33	Not tested	0
	Lee et al.^10^	39	United Kingdom, Italy	69	21	5
	Pardanani et al.^34^	603	United States, Italy	58	Not tested	8.1

Note: Please see the full reference list of Dicks MJ, Nell E-M, Swanepoel C, Abdullah I, Chapanduka ZC. Mutational landscape of classical myeloproliferative neoplasms in the Western Cape Province, South Africa. Afr J Lab Med. 2026;15(1), a2965. https://doi.org/10.4102/ajlm.v15i1.2965.

*CALR, calreticulin* gene; *JAK2, Janus kinase 2* gene; *MPL*, thrombopoietin receptor gene.

Triple-negative ET and PMF were relatively infrequent in our patients at only 6%, which is less than the 10% to 15% reported in international studies.^10^ The number of triple-negative patients in this study may be higher than reported if all the ET and PMF patients who tested negative for *JAK2* p.V617F were also tested for other variants, namely those affecting *CALR* and *MPL*.

Our study showed that patients with PMF were statistically more likely to present with a higher haemoglobin level if they were positive for the *JAK2* p.V617F variant. The *JAK2* p.V617F positive PMF patients were also significantly older at presentation than the *JAK2* p.V617F negative group. These findings correlate with the international literature.^35^ Haemoglobin level and age in PMF were the only phenotypic parameters where a statistically significant difference was associated with a specific variant, despite the fact that laboratory features of *JAK2* p.V617F have been distinguished from those of other variants in PV, ET and PMF.^8,35,36^ Future studies on prognosis of different variants in PMF are required in a South African context.

### Limitations

A limitation was that there were 31 patients who were *JAK2* p.V617F positive who did not meet the criteria for PV and did not have a diagnostic bone marrow examination. They were excluded from analysis, limiting the characterisation of *JAK2* p.V617F MPNs, but also highlighting that investigation of cytoses may be incomplete in our setting. Another limitation was that Sanger sequencing might not be able to detect mutant alleles below 10% to 15%, which could lead to false-negative results.^37^ The *MPL, CALR* and *JAK2* exon 12 variants should be investigated with more sensitive techniques in future South African MPN studies to validate these findings.^38^

### Conclusion

In conclusion, this study highlights the frequency of the MPN subtypes and the mutational landscape of MPNs in a South African hospital. We found no *MPL* variants among the ET and PMF patients tested and no *JAK2* exon 12 variants among the PV patients tested. Polycythaemia vera and PMF were common, while ET was less frequent. This study has thus demonstrated that the proportion of MPN subtypes and mutation frequency in subtypes differ from international studies. Patient outcomes may also differ. Future studies exploring survival based on driver mutations within the South African context could prove valuable.
